# Activation of Peroxisome Proliferator-Activated Receptor *γ* by Rosiglitazone Inhibits Lipopolysaccharide-Induced Release of High Mobility Group Box 1

**DOI:** 10.1155/2012/352807

**Published:** 2012-12-20

**Authors:** Jung Seok Hwang, Eun Sil Kang, Sun Ah Ham, Taesik Yoo, Hanna Lee, Kyung Shin Paek, Chankyu Park, Jin-Hoi Kim, Dae-Seog Lim, Han Geuk Seo

**Affiliations:** ^1^Department of Animal Biotechnology, Konkuk University, Seoul 143-701, Republic of Korea; ^2^Department of Nursing, Semyung University, Jecheon 390-711, Republic of Korea; ^3^Department of Applied Bioscience, College of Life Science, CHA University, Seongnam 463-712, Republic of Korea

## Abstract

Peroxisome proliferator-activated receptors (PPARs) are shown to modulate the pathological status of sepsis by regulating the release of high mobility group box 1 (HMGB1), a well-known late proinflammatory mediator of sepsis. Ligand-activated PPARs markedly inhibited lipopolysaccharide- (LPS) induced release of HMGB1 in RAW 264.7 cells. Among the ligands of PPAR, the effect of rosiglitazone, a specific ligand for PPAR*γ*, was superior in the inhibition of HMGB1 release induced by LPS. This effect was observed in cells that received rosiglitazone before LPS or after LPS treatment, indicating that rosiglitazone is effective in both treatment and prevention. Ablation of PPAR*γ* with small interfering RNA or GW9662-mediated inhibition of PPAR*γ* abolished the effect of rosiglitazone on HMGB1 release. Furthermore, the overexpression of PPAR*γ* markedly potentiated the inhibitory effect of rosiglitazone on HMGB1 release. In addition, rosiglitazone inhibited LPS-induced expression of Toll-like receptor 4 signal molecules, suggesting a possible mechanism by which rosiglitazone modulates HMGB1 release. Notably, the administration of rosiglitazone to mice improved survival rates in an LPS-induced animal model of endotoxemia, where reduced levels of circulating HMGB1 were demonstrated. Taken together, these results suggest that PPARs play an important role in the cellular response to inflammation by inhibiting HMGB1 release.

## 1. Introduction

High mobility group box 1 (HMGB1) is a highly conserved nonhistone nuclear protein that exhibits diverse functions according to its cellular location. In the intracellular compartment, it participates in a number of fundamental cellular processes such as transcription, replication, and DNA repair [1]. In addition to its intracellular functions, extracellular HMGB1 plays an important role in inflammatory responses when actively secreted from stressed cells [2]. Proinflammatory properties of HMGB1 as a crucial cytokine were first documented in a report demonstrating that HMGB1 is actively secreted by activated macrophages, serving as a late mediator of lethality in a mouse model of sepsis [3]. Furthermore, circulating HMGB1 levels were elevated with delayed fashion in the mouse model and in patients with sepsis characterized by overwhelming inflammatory and immune responses, leading to tissue damage, multiple-organ failure and death [3–5]. Recent reports indicated that HMGB1 is a late mediator of sepsis, acting as a key regulator in acute and chronic inflammation [2, 3]. In fact, the administration of anti-HMGB1 antibodies or inhibitors, such as ethyl pyruvate and nicotine, significantly protected mice from LPS-induced acute tissue injury and lethal endotoxemia [3, 4, 6–8]. Notably, these reagents against HMBG1 conferred cellular protection to delayed endotoxin lethality, even when applied at a time after the acute-phase cytokine responses had peaked and resolved [3, 6, 8, 9].

Peroxisome proliferator-activated receptors (PPARs), members of the nuclear hormone receptor family, are ligand-activated transcription factors with multiple biological functions [10, 11]. Three different PPAR isoforms have been identified, PPAR*α* (NR1C1), PPAR*β*/*δ* (NR1C2), and PPAR*γ* (NR1C3), and are encoded by different genes that show substantial amino acid similarity, especially within the DNA and ligand-binding domains [11]. All PPARs act as heterodimers with the retinoid X receptor (RXR) and exhibit pleiotropic effects in the regulation of lipid and glucose metabolism, as well as cellular differentiation and proliferation [10–12]. Recently, there has been a great deal of interest in the involvement of PPARs in inflammatory processes [13]. PPAR ligands inhibit the expression of inflammatory genes and can negatively interfere with proinflammatory transcription factor-signaling pathways in vascular and inflammatory cells [14–16]. Furthermore, PPAR levels are differentially regulated in a variety of inflammatory disorders in human, indicating that ligands for PPAR represent new promising therapies for the treatment of diseases associated with inflammation [14].

Although PPARs have shown anti-inflammatory effects in monocyte/macrophages and vascular cells [14–16], little is known about their involvement in the endotoxin-mediated release of HMGB1. Here, we demonstrate that PPARs are involved in the regulation of LPS-induced HMGB1 release in RAW 264.7 cells, and the administration of rosiglitazone, a specific ligand for PPAR*γ*, attenuated endotoxin lethality by inhibiting HMGB1 release in a mouse model of sepsis.

## 2. Materials and Methods

### 2.1. Materials

GW501516, WY-14643, and GW9662 were obtained from Calbiochem (La Jolla, CA, USA). 5-[[4-(2-[methyl-2-pyridinylamino]ethoxy)phenyl]methyl]-2,4-thiazolidinedione (rosiglitazone) was obtained from Cayman Chemical Company (Ann Arbor, MI, USA). Polyclonal antibodies specific for PPAR*α*, PPAR*β*/*δ*, PPAR*γ*, monocyte chemoattractant protein-1 (MCP-1), tumor necrosis factor-*α* (TNF-*α*), macrophage inflammatory protein-1*β* (MIP-1*β*), and horseradish peroxidase (HRP)-conjugated IgG were supplied by Santa Cruz Biotechnology (Santa Cruz, CA, USA). Rabbit polyclonal antibody specific for *β*-actin, lipopolysaccharide (LPS, *Escherichia coli* 0111:B4), Polyinosinic-polycytidylic acid (Poly (I:C)), and Ponceau S solution were purchased from Sigma-Aldrich Co. (St. Louis., MO, USA). Monoclonal antibodies specific for HMGB1, phospho-I*κ*B*α*, inducible nitric oxide synthase (iNOS), and myeloid differentiation primary responses gene 88 (MyD88) were purchased from Epitomics (Burlingame, CA, USA) and BD Bioscience (San Jose, CA, USA), respectively. TIR-domain-containing adaptor-inducing interferon-*β* (TRIF) was purchased from abcam (Cambridge, UK). Other reagents were of the highest grade available.

### 2.2. Cell Culture and Stimulation

RAW 264.7 cells, a murine macrophage-like cell line, were obtained from American Type Culture Collection (Manassas, VA, USA). Cells were maintained in Dulbecco's modified Eagle's medium (DMEM) containing 100 U/mL penicillin and 100 *μ*g/mL streptomycin, supplemented with 10% heat-inactivated fetal bovine serum at 37°C, under an atmosphere of 95% air and 5% CO_2_. RAW 264.7 cells (2 × 10^6^ cells) were plated in 60 mm culture dishes. At 60% confluency, the cells were incubated with serum-free DMEM medium for 24 h and then stimulated with LPS (100 ng/mL) in the presence or absence of indicated reagents.

### 2.3. Western Blot Analysis

Cells treated with the indicated reagents were washed with ice-cold PBS and lysed in PRO-PREP Protein Extraction Solution (iNtRON Biotechnology, Seoul, Korea). An aliquot of the cell lysate was subjected to SDS-polyacrylamide gel electrophoresis (SDS-PAGE) and transferred onto a Hybond-P^+^ polyvinylidene difluoride membrane (Amersham Biosciences UK Ltd., UK). Membranes were blocked overnight at 4°C, with 5% nonfat milk in Tris-buffered saline (TBS) containing 0.1% Tween 20. Membranes were then incubated overnight at 4°C, with the indicated specific antibodies in TBS containing 1% BSA and 0.05% Tween 20. Finally, membranes were incubated for 2 h at room temperature with peroxidase-conjugated goat antibody diluted 1 : 3000. After extensive washing in TBS containing 0.1% BSA and 0.1% Tween 20, immuno-reactive bands were detected using West-ZOL Plus (iNtRON Biotechnology, Seoul, Republic of Korea).

### 2.4. Determination of HMGB1

An equal aliquot of conditioned culture media from an equal number of RAW 264.7 cells was used to determine the amount of HMGB1 released into culture media. Equal volumes of conditioned culture media were mixed with 80% ice-cold acetone and incubated at −20°C for 1 h. The protein pellet was precipitated following centrifugation at 16,000 g for 10 min at 4°C. After washing with 80% ice-cold acetone, the pellets were resuspended in SDS-PAGE sample buffer and subjected to Western blot analysis.

### 2.5. Construction of Short Hairpin (sh)RNA against PPAR*γ* and Gene Silencing

Two complementary 55-mer siRNA template oligonucleotides, encoding mouse PPAR*γ* short hairpin (sh)RNA with *Bam*HI-*Hind*III overhangs, were designed to knock down PPAR*γ*. The oligonucleotides used were (sense) 5′-GATCCGGATGCAAGGGTTTCTTCC TTCAAGAGAGGAAGAAACCCTTGCATCCTTA-3′ and (anti-sense) 5′-AGCTT AAGGATGCAAGGGTTTCTTCCTCTCTTGAAGGAAGAAACCCTTGCATCCG-3′. The oligonucleotides were then annealed by incubating the mixed oligonucleotides in a PCR thermocycler, using the following profile: 90°C for 3 min, followed by 37°C for 60 min. Annealed oligonucleotides were cloned into the *Bam*HI-*Hind*III-digested expression vector pSilencer 4.1-CMV hygro plasmid (Ambion, Austin, TX, USA). The same vector encoding a single hairpin siRNA sequence not found in the mouse database was constructed and used as a scrambled shRNA control. All DNA oligonucleotides were synthesized by Cosmo Co., Ltd. (Seoul, Republic of Korea). The sequence of the oligonucleotide (5′-AAGGATGCAAGGGTTTCTTCC-3′) was targeted to the PPAR*γ* sequence corresponding to positions 547–564 within the PPAR*γ* mRNA. Transfected RAW 264.7 cells were selected with 100 *μ*g/mL hygromycin, and the efficiency of knockdown was confirmed by Western blot. Small interfering (si)RNA study was performed as described previously [15].

### 2.6. Plasmid Construction

The mammalian expression vector pcDNA3.1-PPAR*γ* was constructed as described previously [17].

### 2.7. Real-Time PCR Analysis

Total RNA was isolated using TRIzol reagent (Invitrogen, Carlsbad, CA, USA), and reverse transcribed into cDNA by TOPscript RT DryMIX kit (Enzynomics, Seoul, Republic of Korea). Equal amounts of cDNA were diluted, amplified by real-time PCR using Rotor Gene RG-3000 (Corbett life Science, Sydney, Australia) in a 10 *μ*L reaction volume containing 1 x SYBR PCR master mix (QIAGEN, Valencia, CA, USA) and 10 *μ*M primers. After an initial denaturation step for 5 min at 95°C, conditions for cycling were 40 cycles of 10 s at 95°C, 10 s at 58.5°C, and 10 s at 72°C. For normalization of each sample, GAPDH primers were used to measure the amount of GAPDH cDNA. The primers used as follows: MyD88, forward 5′-GGAGATGATCCGGCAACTAGAA-3′; reverse 5′-ATTAGCTCGCTGGCAATGGA-3′; TRIF, forward 5′-TTCCAGCCACTCCATTCTCATC-3′; reverse 5′-GTAACGTATGTCCCCAACTCCA-3′; GAPDH, forward 5′-CATGGCCTTCCGTGTTCCTA-3′; reverse 5′-CCTGCTTCACCACCTTCTTGAT-3′. The fold change in target gene cDNA relative to the GAPDH control was determined by delta delta CT method [18].

### 2.8. Animal Model of Endotoxemia and Serum Analysis

All animal studies were approved by the Institutional Animal Care Committee of Konkuk University. Endotoxemia was induced in BALB/c mice (male, 6-7 weeks, 20–25 g) by intraperitoneal injection of bacterial endotoxin (10 mg/kg, *Escherichia coli* LPS 0111:B4), as described previously [3, 6]. Briefly, BALB/c mice were obtained from Koatech (Pyeongtaek, Korea) and housed in a pathogen-free environment. Standard sterilized laboratory diet and water were available *ad libitum* under controlled environmental conditions, with a 12 h light/dark cycle (light on 06:00). BALB/c mice were randomly assigned to one of four groups: injection of LPS (10 mg/kg), injection of LPS (10 mg/kg) plus rosiglitazone (10 mg/kg), injection of LPS (10 mg/kg) plus rosiglitazone (10 mg/kg) plus GW9662 (1 mg/kg), or injection of GW9662 (1 mg/kg) alone. Another group of BALB/c mice were treated with rosiglitazone (10 mg/mL) after LPS (10 mg/kg) infusion. Mortality was recorded for up to 2 weeks after LPS injection to ensure that no additional late deaths occurred. For measurement of plasma HMGB1 levels, BALB/c mice were subjected to sepsis by LPS injection in the presence or absence of rosiglitazone as described above. After 20 h, blood was collected, allowed to clot for 2 h at room temperature, and then centrifuged for 20 min at 1,500 g. The levels of circulating HMGB1 in serum were determined by Western blot analysis.

### 2.9. Statistical Analysis

Data are expressed as means ± SE. Statistical significance was determined by Student's *t*-test or ANOVA with a post hoc Bonferroni test. A value of *P* < 0.05 was considered statistically significant.

## 3. Results

### 3.1. Activation of PPARs by Ligand Inhibits LPS-Induced Release of HMGB1 in RAW 264.7 Cells

**Figure 1:**
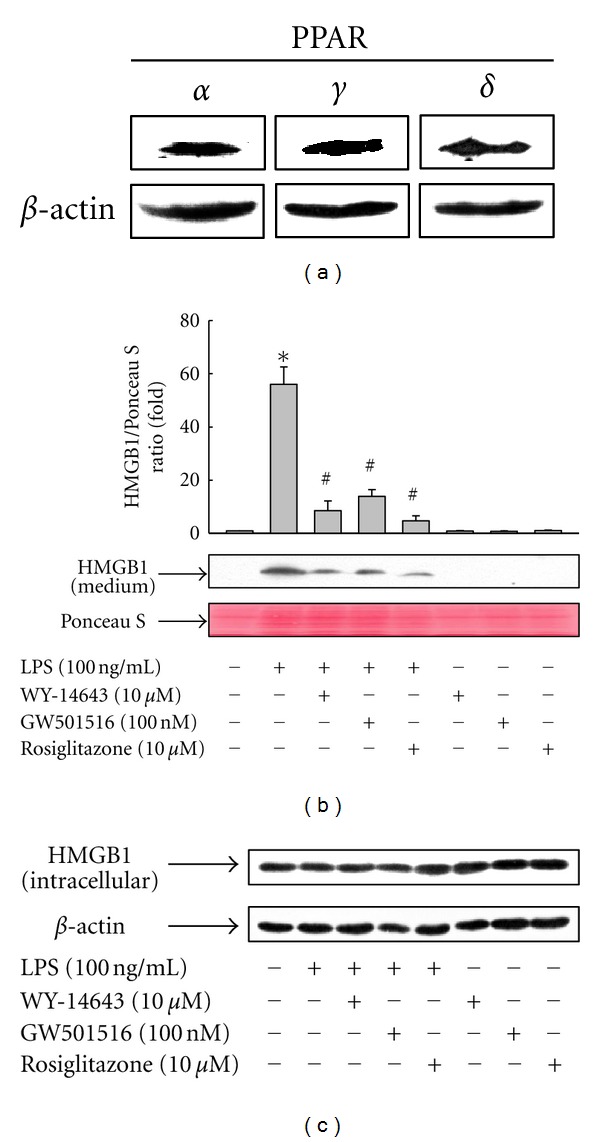
Activation of PPARs by ligand inhibits LPS-induced release of HMGB1 in RAW 264.7 cells. (a) Cells were harvested and the expression levels of PPARs were detected by using Western blot with indicated antibodies, as described in Section 2. (b) Cells grown to 60% confluency were incubated with serum-free medium for 24 h and then stimulated with LPS in the presence or absence of ligands for 24 h. Equal volumes of conditioned media were subjected to Western blot analysis. Ponceau S staining was used as a loading control. (c) At the same time, total protein was extracted, fractionated by electrophoresis, and immunoblotted with the indicated antibodies. Representative blots and densitometric measurements from three independent experiments are shown. The results are expressed as the means ± S.E. (*n* = 3). *P < 0.01 compared to untreated group; ^#^ *P* < 0.01 compared to LPS-treated group.

To investigate whether PPARs exhibit biological functions in RAW 264.7 cells, constitutive expression of PPARs was examined. Expression of three PPAR isoforms was observed (Figure 1(a)), suggesting that all of the PPAR isoforms may be biologically active in RAW 264.7 cells. To determine whether the activation of PPARs by ligand affects endogenous HMGB1 endogenous expression or release, RAW 264.7 cells were treated with LPS for 24 h, and the release of HMGB1 was measured. Levels of secreted HMGB1 were significantly increased upon LPS treatment, and this increase was markedly suppressed in the presence of PPAR ligands, suggesting the involvement of PPARs in the inhibition of LPS-induced HMGB1 release (Figure 1(b)). Among the ligands for PPAR, rosiglitazone, a specific ligand for PPAR*γ*, was superior to others in the inhibition of LPS-induced HMGB1 release. In contrast to that of secreted levels, neither LPS nor ligands of PPARs affected the expression levels of endogenous HMGB1 (Figure 1(c)). These results indicate that PPARs are involved in the regulation of LPS-induced HMGB1 release, but not in the regulation of HMGB1 expression. Under the concentrations of ligands used in these experiments, cells retained good viability within the experimental time frames used, as determined by trypan blue exclusion method (see Supplementary Figure 1 available online at doi:10.1155/2012/352807).

### 3.2. Rosiglitazone Inhibits Poly (I:C)-Induced HMGB1 Release in RAW 264.7 Cells

**Figure 2:**
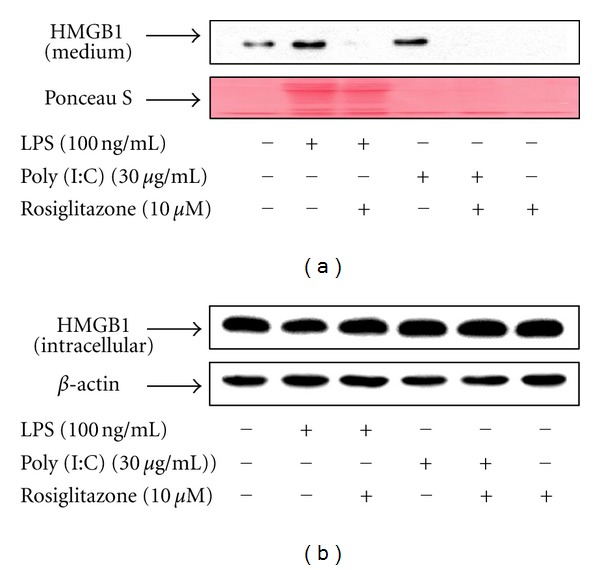
Rosiglitazone inhibits Poly (I:C)-induced HMGB1 release in RAW 264.7 cells. (a) Cells grown to 60% confluency were incubated with serum-free medium for 24 h and then stimulated with LPS and/or Poly (I:C) in the presence or absence of rosiglitazone for 24 h. Equal volumes of conditioned media were subjected to Western blot analysis for the detection of HMGB1. Ponceau S staining was used as a loading control. (b) At the same time, total protein was extracted, fractioned by electrophoresis, and immunoblotted with the indicated antibodies. The results shown are representative of three independent experiments.

To examine whether rosiglitazone has a specific effect against LPS stimulation in the inhibition of HMGB1 release, RAW 264.7 cells were stimulated with Poly (I:C), a well-known ligand of Toll-like receptor (TLR) 3, in the presence or absence of rosiglitazone for 24 h. Rosiglitazone significantly inhibited Poly (I:C)-induced HMGB1 release, but not affect expression levels of HMGB1 (Figure 2), indicating that effect of rosiglitazone on the inhibition of HMGB1 release is not limited to the LPS.

### 3.3. Rosiglitazone Also Attenuates LPS-Induced Release of HMGB1 in RAW 264.7 Cells When Administered Following LPS Treatment

**Figure 3:**
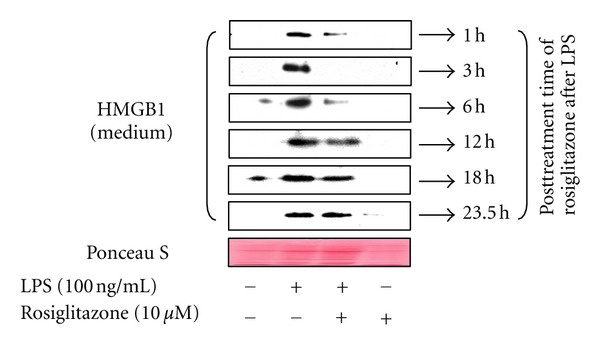
Administration of rosiglitazone post-LPS treatment also attenuates LPS-induced release of HMGB1 in RAW 264.7 cells. Cells were grown to 60% confluency, incubated with serum-free medium for 24 h, and then treated with LPS. Rosiglitazone was administered at the indicated time points post-LPS treatment for 24 h. Conditioned medium was subjected to Western blot analysis for the determination of HMGB1 levels. Ponceau S staining was used as a loading control. The results shown are representative of three independent experiments.

Because administering rosiglitazone to cells prior to treatment with LPS was effective in the inhibition of LPS-induced release of HMGB1, the effect of rosiglitazone when supplied at time points following LPS treatment was examined. When cells were treated with LPS, an increase in the level of released HMGB1 was detected at 24 h, and this increase was markedly reduced by supplying rosiglitazone to cells following LPS treatment. This effect was observed in cells when rosiglitazone was administered up to 6 h after LPS treatment and also, to a lesser extent, in cells receiving rosiglitazone up to 18 h after LPS treatment (Figure 3), suggesting that rosiglitazone could be useful in treatment, as well as in the prevention of HMGB1 release.

Since PPAR*γ* was reported to mediate inflammatory responses by inhibiting proinflammatory cytokines [19, 20], the effects of rosiglitazone on the secretion of inflammatory cytokines such as TNF-*α*, MCP-1, and MIP-1*β* were examined. A marked increase in the levels of MCP-1, MIP-1*β*, and TNF-*α* was observed in RAW 264.7 cells treated with LPS for 24 h, whereas simultaneous administration of rosiglitazone clearly reduced the effect of LPS on the level of MCP-1 and MIP-1*β*, but not on the level of TNF-*α* (see Supplementary Figure 2). Finally, the effect of rosiglitazone on the expression of inducible nitric oxide synthase (iNOS) was examined. The LPS-induced upregulation of iNOS expression was markedly attenuated in the presence of rosiglitazone, corroborating the effects of rosiglitazone observed in the regulation of inflammation induced by LPS (see Supplementary Figure 3).

### 3.4. Rosiglitazone-Mediated Inhibition of HMGB1 Release Is Dependent on PPAR*γ* in RAW 264.7 Cells Treated with LPS

**Figure 4:**
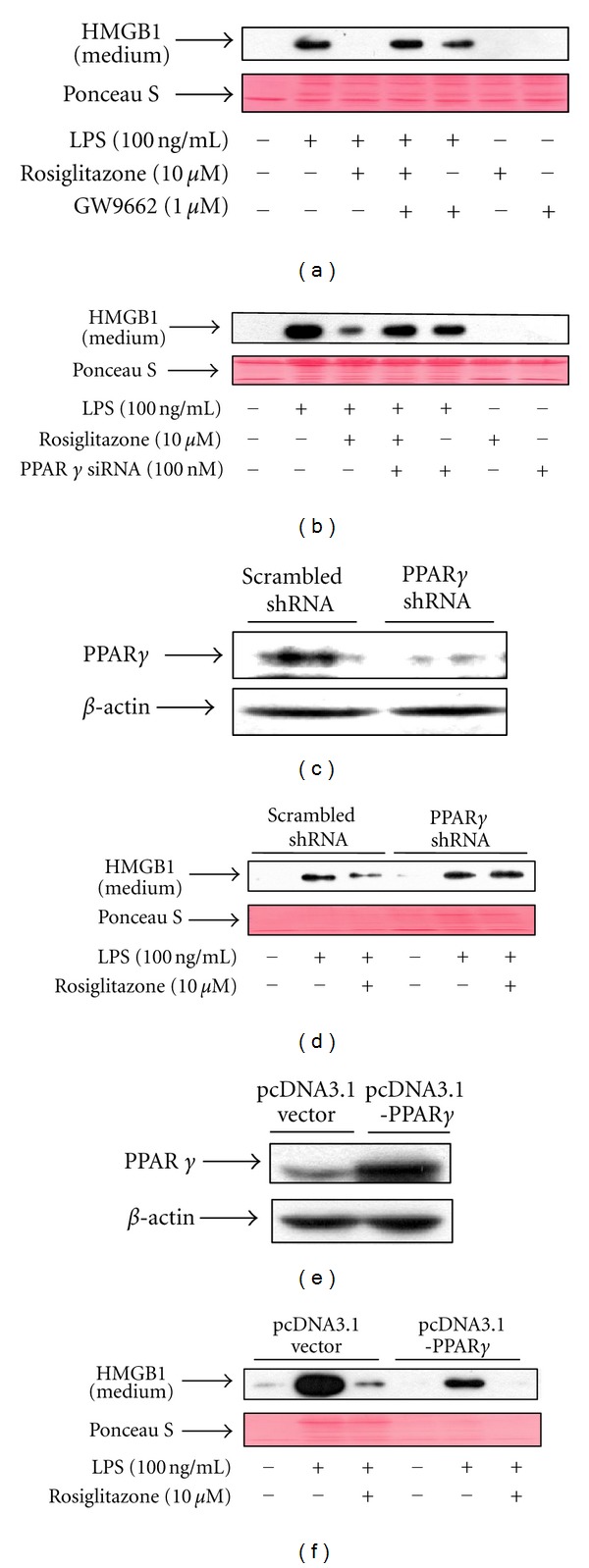
PPAR*γ* regulates LPS-induced release of HMGB1 in RAW 264.7 cells. (a) Cells pretreated for 1 h with GW9662 were stimulated with LPS in the presence or absence of rosiglitazone for 24 h. Conditioned medium was collected and subjected to Western blot analysis for determination of HMGB1 levels. (b) Cells transfected with PPAR*γ* siRNA for 38 h were incubated with serum-free medium for 24 h, and then treated with LPS in the presence or absence of rosiglitazone for 24 h. Equal volumes of conditioned media were subjected to Western blot analysis. (c) Cells were transfected with a vector encoding one hairpin siRNA against PPAR*γ* or encoding a scrambled shRNA control. Stable transfectants were selected with 100 *μ*g/mL hygromycin, and the expression levels of PPAR*γ* were determined by Western blot analysis. (d) Cells expressing PPAR*γ* shRNA or scrambled control shRNA were treated with LPS in the presence or absence of rosiglitazone for 24 h. Conditioned medium was subjected to Western blot analysis for the determination of HMGB1 levels. (e) Cells transfected with pcDNA3.1-PPAR*γ*, or pcDNA3.1 vector for 48 h were harvested and subjected to Western blot analysis with indicated antibodies. (f) Cells transfected with pcDNA 3.1 or pcDNA3.1-PPAR*γ* for 48 h were incubated with serum-free medium for 24 h and then stimulated with LPS in the presence or absence of rosiglitazone for 24 h. Equal volumes of conditioned media were subjected to Western blot analysis for the detection of HMGB1. Ponceau S staining was used as a loading control. The results shown are representative of three independent experiments.

To examine the role of PPAR*γ* in rosiglitazone-mediated inhibition of HMGB1 release induced by LPS, RAW 264.7 cells were treated with siRNA against PPAR*γ* or GW9662, an irreversible inhibitor of PPAR*γ* [21]. In LPS-treated RAW 264.7 cells, the addition of PPAR*γ* siRNA or GW9662 almost completely abolished the rosiglitazone-mediated inhibition of HMGB1 release (Figures 4(a) and 4(b)). To further ascertain the effect of endogenous PPAR*γ* on LPS-induced HMGB1 release, knockdown or overexpression of PPAR*γ* using a specific shRNA or vector-host systems, respectively, was carried out. RAW 264.7 cells stably expressing PPAR*γ* shRNA were established and shown to exhibit a reduced level of PPAR*γ* expression, whereas PPAR*γ* expression in cells transfected with a vector expressing scrambled shRNA was unaffected (Figure 4(c)). This PPAR*γ*-shRNA-mediated downregulation of PPAR*γ* counteracted the inhibitory effects of rosiglitazone on the HMGB1 release induced by LPS (Figure 4(d)). Furthermore, overexpression of PPAR*γ* had more pronounced effects in HMGB1 release (Figures 4(e) and 4(f)). These data clearly indicate that PPAR*γ* directly regulates LPS-induced HMGB1 release in RAW 264.7 cells.

### 3.5. Rosiglitazone Inhibits TLR4 Signal Pathway Stimulated by LPS

**Figure 5:**
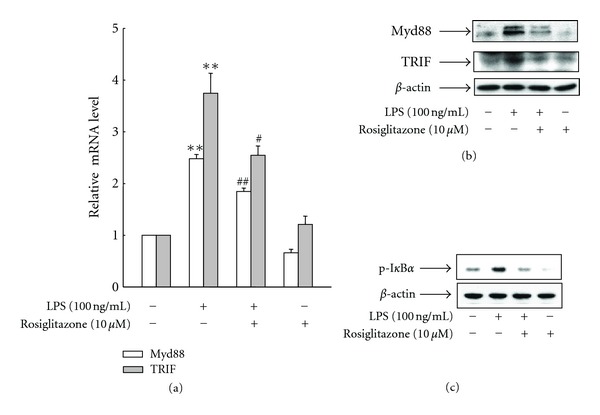
Rosiglitazone inhibits LPS-induced TLR4 signal pathway in RAW 264.7 cells. (a) Cells incubated with serum-free medium for 24 h were treated with LPS in the presence or absence of rosiglitazone for 6 h. The mRNA levels of indicated genes were determined by real-time PCR using SYBR Green. GAPDH was used as an internal standard. (b) Cells were incubated in serum-free medium for 24 h and then stimulated with LPS in the presence or absence of rosiglitazone for 9 h. Total protein was extracted, fractionated by electrophoresis, and immunoblotted with the indicated antibodies. (c) Cells incubated with serum-free medium for 24 h were treated with LPS in the presence or absence of rosiglitazone for 1 h. An aliquot of protein was immunoblotted with phospho-I*κ*B*α* (p-I*κ*B*α*) and *β*-actin antibodies. The results are expressed as the means ± S.E. (*n* = 3). **P < 0.01 compared to untreated group; ^#^ *P* < 0.05, ^##^ *P* < 0.01 compared to LPS-treated group.

Since TLR4 is involved in the regulation of LPS-induced HMGB1 release [22, 23], we examined whether rosiglitazone affects TLR4 signal pathway in LPS-treated RAW 264.7 cells. Expression of MyD88 and TRIF, key adaptor molecules of TLR4, was increased in RAW264.7 cells treated with LPS for 6 h, whereas simultaneous administration of rosiglitazone significantly reduced the levels of MyD88 and TRIF (Figures 5(a) and 5(b)). In addition, rosiglitazone also significantly inhibited the LPS-induced phosphorylation of I*κ*B*α* (Figure 5(c)), indicating that the activation of PPAR*γ* by rosiglitazone modulates TLR4 signal pathway by inhibiting the LPS-induced expression of MyD88/TRIF, and consequent blocking its effector NF-*κ*B.

### 3.6. Rosiglitazone Attenuates Endotoxin-Induced Lethality through PPAR*γ*-Mediated Inhibition of HMGB1 Release

**Figure 6:**
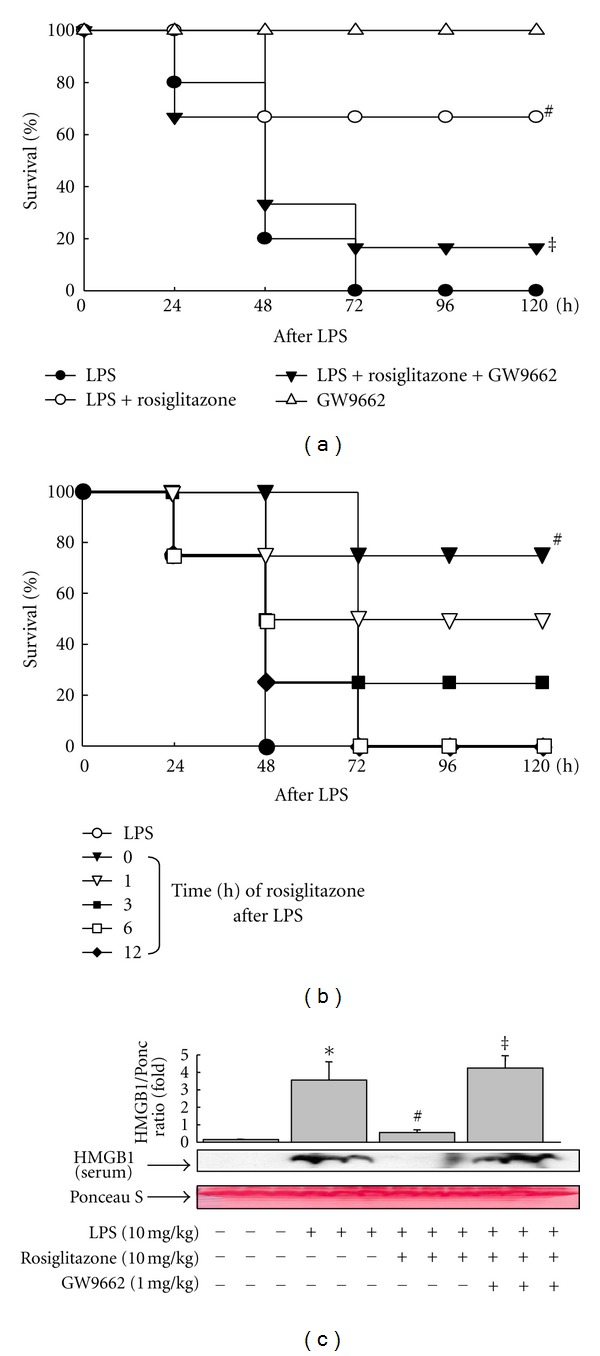
Rosiglitazone prevents endotoxin lethality by attenuating HMGB1 release *in vivo*. (a) BALB/c mice (*n* = 10-11 per group) were injected with a single dose of rosiglitazone (10 mg/kg, i.p.) with or without GW9662 (1 mg/kg, i.p.), followed 30 min later by a lethal infusion of endotoxin (LPS, 10 mg/kg, i.p.). (b) BALB/c mice (*n* = 4 per group) infused with endotoxin (LPS, 10 mg/kg, i.p.) were treated with rosiglitazone (10 mg/kg, i.p.) 0, 1, 3, 6, and 12 h later. Survival was monitored daily for up to 2 weeks. (c) In a parallel group of rosiglitazone-administered mice, circulating levels of HMGB1 were detected by Western blot analysis using sera prepared from samples collected at 20 h post-LPS injection. Representative blots from four independent experiments and densitometric measurements are shown. Ponceau S staining was used as a loading control. The results are expressed as the means ± S.E. (*n* = 3). *P < 0.01 compared to the untreated group; ^#^ *P* < 0.01 compared to the LPS-treated group; ^‡^ *P* < 0.05 compared to the LPS plus rosiglitazone-treated group.

To further investigate the *in vivo* relevance of these *in vitro* results, an initial evaluation of rosiglitazone as a therapeutic agent was performed using a standard model of murine endotoxemia. Injection of LPS dramatically increased the mortality of mice, whereas the administration of rosiglitazone prior to LPS treatment significantly improved the survival rates (Figure 6(a)). This effect of rosiglitazone was significantly reduced in the presence of GW9662, indicating the involvement of PPAR*γ* in the rosiglitazone-mediated improvement of survival rates. Late deaths in rosiglitazone and/or GW9662-administered animals were not observed during the 2 weeks following endotoxin injection (data not shown), indicating that administration of rosiglitazone conferred protection to mice against lethal endotoxemia. Furthermore, the posttreatment of rosiglitazone after LPS also improved the survival rates until after 3 h (Figure 6(b)), demonstrating that rosiglitazone has an extended therapeutic window. Because endotoxin lethality is corelated to HMGB1 release [3, 4, 6–8], the effects of rosiglitazone on the level of circulating HMGB1 in blood were determined. HMGB1 blood levels significantly increased by LPS injection, whereas the administration of rosiglitazone almost completely abolished the release of HMGB1 into the blood, which was reversed by the presence of GW9662 (Figure 6(c)). These results indicated that rosiglitazone prevents endotoxin lethality* in vivo* by blocking HMGB1 release.

## 4. Discussion

In the present study, ligand-activated PPARs were shown to inhibit LPS-induced release of HMGB1, indicating a role for PPARs as regulatory molecules of HMGB1 release. Rosiglitazone, a specific ligand for PPAR*γ*, is superior to other PPAR ligands in the inhibition of HMGB1 release induced by the presence of LPS. This is the first report demonstrating that ligand-activated PPARs inhibit LPS-stimulated HMGB1 release in RAW 264.7 cells. A recent report demonstrated that telmisartan, a non-selective ligand for PPAR*γ*, protected against postischemic injury by partially inhibiting the inflammatory reaction via a PPAR*γ*-dependent HMGB1-inhibiting mechanism [24]. On the other hand, a different line of investigation showed that PPAR*γ* agonist troglitazone inhibited HMGB1 expression in endothelial cells [25]. This is in contrast to the present findings that endogenous HMGB1 expression was unaffected by ligand activation of PPARs, whereas ligand activation of PPARs caused HMGB1 release in RAW 264.7 cells treated with LPS. No conclusive data are available at present although the induction of HMGB1 expression by PPAR ligands was not observed even at ligand concentrations as high as 100 *μ*M (data not shown). However, the possibility remains that this discrepancy could be due to the different ligands or different cell types investigated in these reports. Further studies are, therefore, necessary to clarify the role of PPAR*γ* in the regulation of HMGB1 induced by LPS.

Of particular interest is the possibility that ligand activation of PPAR*γ* may participate in the pathophysiology of sepsis. The ligand-activated PPAR*γ* caused a marked attenuation in the LPS or Poly (I:C)-induced release of HMGB1. Moreover, the administration of rosiglitazone either pre- or post-LPS treatment inhibited LPS-induced release of HMGB1, indicating that HMGB1 regulation by rosiglitazone is effective in both the treatment and prevention of HMGB1 release. Furthermore, the administration of PPAR*γ* ligand decreased the endotoxin-induced lethality of LPS in mice and reduced levels of circulating HMGB1, indicating that the effects of PPAR*γ* on septic shock are HMGB1 dependent. This finding is in line with previous studies in which high levels of HMGB1 were demonstrated in patients with severe sepsis and in animals models of endotoxemia [3, 4, 26, 27], suggesting that HMGB1 may play a crucial role in the process of sepsis. PPAR*γ* has also been reported to mediate inflammatory responses by inhibiting proinflammatory cytokines, such as TNF-*α*, interleukin (IL)-6, and iNOS [19, 20, 28]. However, little is known about the regulatory role of PPAR*γ* in HMGB1 release, a late phase mediator of inflammatory responses *in vitro* and *in vivo*. Accordingly, these results suggested that, under pathological conditions, PPAR*γ* may play a major role as an anti-inflammatory molecule through the inhibition of early and late phase mediators.

The mechanism by which PPAR*γ* controls LPS-induced HMGB1 release remains unclear. As a transcription factor, PPAR*γ* primarily regulates gene expression through its binding with its heterodimeric partner RXR to a specific recognition site, termed the peroxisome proliferator response element (PPRE), in the promoter region of a target gene [29]. However, the consensus PPRE motif was not identified in either the rat or human promoter regions of HMGB1 [25]. Thus, the regulatory mechanism of PPAR*γ* for HMGB1 release may be a result of a secondary effect causing modifications of HMGB1 related to translocation, such as acetylation or phosphorylation [30, 31]. Posttranslational modification of HMGB1 appears to be critical for HMGB1 release [30, 31]. In fact, it has been reported that HMGB1 is extensively modified, hyperacetylated in LPS-activated monocytes, and released to participate in the inflammatory response [31, 32]. Although our present data showed that TLR4 signal pathway was affected by PPAR*γ* activation, extrapolation of our present data awaits further study to reveal the PPAR*γ*-mediated regulatory mechanisms underlying the release of HMGB1 induced by LPS.

In summary, data presented here demonstrate that PPAR*γ* changes the cellular responses of cells to bacterial endotoxin *in vitro* and *in vivo*, hence supporting the hypothesis that PPAR*γ* is involved in inflammatory processes by attenuating mediators released during exposure to endotoxin. Accordingly, this study provides new insights into the pleiotropic roles of PPAR*γ* via the regulation of HMGB1 release and may lead to a better understanding of the clinical efficacy of rosiglitazone in the treatment of inflammation-related disorders.

## Supplementary Material

Supplementary Figure 1: Effects of PPAR ligands on cell viability. RAW264.7 cells were incubated with various concentrations of WY-14643 (a specific agonist for PPAR**α**), GW501516 (a specific agonist for PPAR**σ**), or rosiglitazone (a specific agonist for PPAR**γ**) for 24 h. Cells were then washed with ice-cold PBS, harvested, and mixed with a 0.4% trypan blue solution. Viable cells in the cell suspension were counted using a hemacytometer. Data are expressed as the means±SE (n=6).
Supplementary Figure 2: Effects of rosiglitazone on the LPS-induced release of MCP-1, MIP-1**β**, and TNF-*α*. RAW264.7 cells grown to 60% confluency were incubated with serum-free medium for 24 h and then stimulated with LPS in the presence or absence of rosiglitazone for 24 h. Equal volumes of conditioned media were subjected to Western blot analysis. Ponceau S staining was used as a loading control.
Supplementary Figure 3: Effects of rosiglitazone on the expression of iNOS. RAW264.7 cells were grown to 60% confluency incubated with serum-free medium for 24 h and then stimulated with LPS in the presence or absence of rosiglitazone for 24 h. Total protein was extracted, fractionated by electrophoresis, and immunoblotted using an iNOS antibody.

## Abbreviations

HMGB1: High mobility group box 1
iNOS: Inducible nitric oxide synthase
LPS: Lipopolysaccharides
MIP-1*β*: Macrophage inflammatory protein-1*β*
MCP-1: Monocyte chemoattractant protein-1
MyD88: Myeloid differentiation primary responses gene 88
Poly (I:C): Polyinosinic-polycytidylic acid
PPAR: Peroxisome proliferator-activated receptor
PPRE: Peroxisome proliferator response element
RXR: Retinoid X receptor
shRNA: Small hairpin RNA
siRNA: Small interfering RNA
TLR: Toll-like receptor
TNF-*α*: Tumor necrosis factor-*α*
TRIF: TIR-domain-containing adaptor-inducing interferon-*β*.

## References

[1] Štros M (2010). HMGB proteins: interactions with DNA and chromatin. *Biochimica et Biophysica Acta*.

[2] Andersson U, Tracey KJ (2010). HMGB1 is a therapeutic target for sterile inflammation and infection. *Annual Review of Immunolgy*.

[3] Wang H, Bloom O, Zhang M (1999). HMG-1 as a late mediator of endotoxin lethality in mice. *Science*.

[4] Yang H, Ochani M, Li J (2004). Reversing established sepsis with antagonists of endogenous high-mobility group box 1. *Proceedings of the National Academy of Sciences of the United States of America*.

[5] Sundén-Cullberg J, Norrby-Teglund A, Rouhiainen A (2005). Persistent elevation of high mobility group box-1 protein (HMGB1) in patients with severe sepsis and septic shock. *Critical Care Medicine*.

[6] Ulloa L, Ochani M, Yang H (2002). Ethyl pyruvate prevents lethality in mice with established lethal sepsis and systemic inflammation. *Proceedings of the National Academy of Sciences of the United States of America*.

[7] Chen G, Li J, Qiang X (2005). Suppression of HMGB1 release by stearoyl lysophosphatidylcholine: an additional mechanism for its therapeutic effects in experimental sepsis. *Journal of Lipid Research*.

[8] Wang H, Liao H, Ochani M (2004). Cholinergic agonists inhibit HMGB1 release and improve survival in experimental sepsis. *Nature Medicine*.

[9] Wang H, Li W, Li J (2006). The aqueous extract of a popular herbal nutrient supplement, Angelica sinensis, protects mice against lethal endotoxemia and sepsis. *Journal of Nutrition*.

[10] Issemann I, Green S (1990). Activation of a member of the steroid hormone receptor superfamily by peroxisome proliferators. *Nature*.

[11] Mangelsdorf DJ, Thummel C, Beato M (1995). The nuclear receptor super-family: the second decade. *Cell*.

[12] Tugwood JD, Issemann I, Anderson RG, Bundell KR, McPheat WL, Green S (1992). The mouse peroxisome proliferator activated receptor recognizes a response element in the 5′ flanking sequence of the rat acyl CoA oxidase gene. *EMBO Journal*.

[13] Harmon GS, Lam MT, Glass CK (2011). PPARs and lipid ligands in inflammation and metabolism. *Chemical Review*.

[14] Moraes LA, Piqueras L, Bishop-Bailey D (2006). Peroxisome proliferator-activated receptors and inflammation. *Pharmacology and Therapeutics*.

[15] Kim HJ, Ham SA, Kim SU (2008). Transforming growth factor-*β*1 is a molecular target for the peroxisome proliferator-activated receptor *δ*. *Circulation Research*.

[16] Chawla A, Barak Y, Nagy L, Liao D, Tontonoz P, Evans RM (2001). PPAR-*γ* dependent and independent effects on macrophage-gene expression in lipid metabolism and inflammation. *Nature Medicine*.

[17] Kim HJ, Woo IS, Kang ES (2006). Identification of a truncated alternative splicing variant of human PPAR*γ*1 that exhibits dominant negative activity. *Biochemical and Biophysical Research Communications*.

[18] Livak KJ, Schmittgen TD (2001). Analysis of relative gene expression data using real-time quantitative PCR and the 2-ΔΔCT method. *Methods*.

[19] Yu JH, Kim KH, Kim H (2008). SOCS 3 and PPAR-*γ* ligands inhibit the expression of IL-6 and TGF-*β*1 by regulating JAK2/STAT3 signaling in pancreas. *International Journal of Biochemistry and Cell Biology*.

[20] Wang CZ, Zhang Y, Li XD (2011). PPAR*γ* agonist suppresses TLR4 expression and TNF-*α* production in LPS stimulated monocyte leukemia cells. *Cell Biochemistry and Biophysics*.

[21] Leesnitzer LM, Parks DJ, Bledsoe RK (2002). Functional consequences of cysteine modification in the ligand binding sites of peroxisome proliferator activated receptors by GW9662. *Biochemistry*.

[22] Yu M, Wang H, Ding A (2006). HMGB1 signals through toll-like receptor (TLR) 4 and TLR2. *Shock*.

[23] Kim JH, Kim SJ, Lee IS (2009). Bacterial endotoxin induces the release of high mobility group box 1 via the IFN-*β* signaling pathway. *Journal of Immunology*.

[24] Haraguchi T, Takasaki K, Naito T (2009). Cerebroprotective action of telmisartan by inhibition of macrophages/microglia expressing HMGB1 via a peroxisome proliferator-activated receptor *γ*-dependent mechanism. *Neuroscience Letters*.

[25] Gao M, Hu Z, Zheng Y (2011). Peroxisome proliferator-activated receptor *γ* agonist troglitazone inhibits high mobility group box 1 expression in endothelial cells via suppressing transcriptional activity of nuclear factor *κ*B and activator protein 1. *Shock*.

[26] Sakamoto Y, Mashiko K, Matsumoto H, Hara Y, Kutsukata N, Yamamoto Y (2007). Relationship between effect of polymyxin B-immobilized fiber and high-mobility group box-1 protein in septic shock patients. *ASAIO Journal*.

[27] Ueno T, Ikeda T, Ikeda K (2011). HMGB-1 as a useful prognostic biomarker in sepsis-induced organ failure in patients undergoing PMX-DHP. *Journal of Surgical Research*.

[28] Tsoyi K, Ha YM, Kim YM (2009). Activation of PPAR-*γ* by carbon monoxide from CORM-2 leads to the inhibition of iNOS but not COX-2 expression in LPS-stimulated macrophages. *Inflammation*.

[29] Jiang C, Ting AT, Seed B (1998). PPAR-*γ* agonists inhibit production of monocyte inflammatory cytokines. *Nature*.

[30] Ju HY, Shin JS (2006). Nucleocytoplasmic shuttling of HMGB1 is regulated by phosphorylation that redirects it toward secretion. *Journal of Immunology*.

[31] Bonaldi T, Talamo F, Scaffidi P (2003). Monocytic cells hyperacetylate chromatin protein HMGB1 to redirect it towards secretion. *EMBO Journal*.

[32] Evankovich J, Cho SW, Zhang R (2010). High mobility group box 1 release from hepatocytes during ischemia and reperfusion injury is mediated by decreased histone deacetylase activity. *Journal of Biological Chemistry*.

