# Supplementary material for: Activation of Peroxisome Proliferator-Activated Receptor γ by Rosiglitazone Inhibits Lipopolysaccharide-Induced Release of High Mobility Group Box 1
Source: Mediators Inflamm. 2012 Dec 20;2012:352807. doi: 10.1155/2012/352807 (PMC3539392; doi:10.1155/2012/352807)

Supplementary figure legends

Supplementary figure 1: Effects of PPAR ligands on cell viability. RAW264.7 cells were incubated with various concentrations of WY-14643 (a specific agonist for PPARα), GW501516 (a specific agonist for PPARδ), or rosiglitazone (a specific agonist for PPARγ) for 24 h. Cells were then washed with ice-cold PBS, harvested, and mixed with a 0.4% trypan blue solution. Viable cells in the cell suspension were counted using a hemacytometer. Data are expressed as the means±SE (n=6).

Supplementary figure 2: Effects of rosiglitazone on the LPS-induced release of MCP-1, MIP-1β, and TNF-α. RAW264.7 cells grown to 60% confluency were incubated with serum-free medium for 24 h and then stimulated with LPS in the presence or absence of rosiglitazone for 24 h. Equal volumes of conditioned media were subjected to Western blot analysis. Ponceau S staining was used as a loading control.

Supplementary Figure 3: Effects of rosiglitazone on the expression of iNOS. RAW264.7 cells were grown to 60% confluency incubated with serum-free medium for 24 h and then stimulated with LPS in the presence or absence of rosiglitazone for 24 h. Total protein was extracted, fractionated by electrophoresis, and immunoblotted using an iNOS antibody.

Supplementary Figure 1

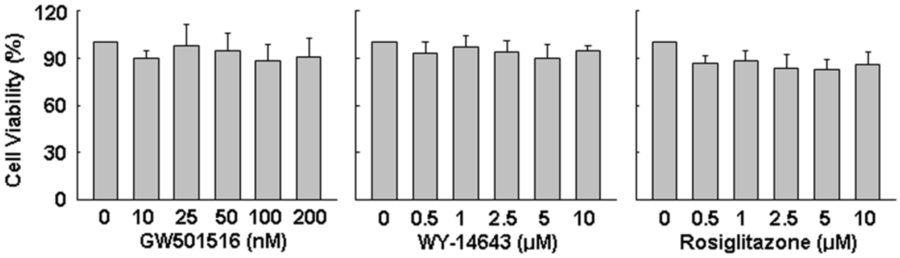

Supplementary Figure 2

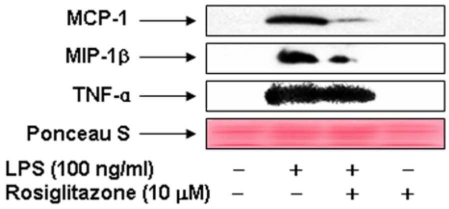

Supplementary Figure 3

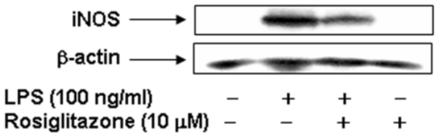

Supplement: Supplementary file 1 — Supplementary Figure 1: Effects of PPAR ligands on cell viability. RAW264.7 cells were incubated with various concentrations of WY-14643 (a specific agonist for PPARα), GW501516 (a specific agonist for PPARσ), or rosiglitazone (a specific agonist for PPARγ) for 24 h. Cells were then washed with ice-cold PBS, harvested, and mixed with a 0.4% trypan blue solution. Viable cells in the cell suspension were counted using a hemacytometer. Data are expressed as the means±SE (n=6). Supplementary Figure 2: Effects of rosiglitazone on the LPS-induced release of MCP-1, MIP-1β, and TNF-α. RAW264.7 cells grown to 60% confluency were incubated with serum-free medium for 24 h and then stimulated with LPS in the presence or absence of rosiglitazone for 24 h. Equal volumes of conditioned media were subjected to Western blot analysis. Ponceau S staining was used as a loading control. Supplementary Figure 3: Effects of rosiglitazone on the expression of iNOS. RAW264.7 cells were grown to 60% confluency incubated with serum-free medium for 24 h and then stimulated with LPS in the presence or absence of rosiglitazone for 24 h. Total protein was extracted, fractionated by electrophoresis, and immunoblotted using an iNOS antibody. [file 352807.f1.pdf]
